# Supplementary material for: Rapid and Easy High-Molecular-Weight Glutenin Subunit Identification System by Lab-on-a-Chip in Wheat (Triticum aestivum L.)
Source: Plants (Basel). 2020 Nov 9;9(11):1517. doi: 10.3390/plants9111517 (PMC7695314; doi:10.3390/plants9111517)
Supplement: Supplementary file 1 [file plants-09-01517-s001.zip › Supplementary Figures and Tables.docx]

Table S1. Molecular weight (MW) and relative protein quantity (RQ) of standard varieties by Lab-on-a-chip. MW and RQ was analyzed by three independent experiments. Values are the mean ± SD.

| Variety Name | HMW-GS subunit | MW(kDa) | RQ(%) |
| --- | --- | --- | --- |
| Jokyung | 8&10 | 133.0 ± 0.54 | 36.6 ± 3.32 |
|  | 1Bx7 | 167.3 ± 0.73 | 23.8 ± 1.07 |
|  | 1Ax1 | 200.6 ± 0.33 | 9.8 ± 2.26 |
|  | 1Dx5 | 210.1 ± 0.29 | 29.8 ± 1.17 |
| Keumgang | 8&10 | 134.0 ± 0.41 | 33.1 ± 1.67 |
|  | 1Bx7 | 167.1 ± 1.00 | 24.0 ± 0.42 |
|  | 1Ax2* | 192.4 ± 0.70 | 13.8 ± 0.90 |
|  | 1Dx5 | 211.4 ± 1.21 | 29.1 ± 0.96 |
| Uri | 1Dy12 | 122.8 ± 1.96 | 24.1 ± 0.88 |
|  | 1By8 | 133.5 ± 1.36 | 16.1 ± 0.55 |
|  | 1Bx7 | 170.0 ± 4.18 | 33.1 ± 3.85 |
|  | 1Dx2.2 | 282.1 ± 7.95 | 26.7 ± 4.05 |
| Petrel | 1Dy10 | 133.7 ± 2.25 | 20.3 ± 0.40 |
|  | 1Dx7 | 177.8 ± 3.16 | 32.4 ± 2.56 |
|  | 1Dx5 | 224.0 ± 1.67 | 47.2 ± 2.86 |
| Seodun | 1Dy12 | 123.3 ± 1.25 | 23.6 ± 2.09 |
|  | 1By8 | 133.9 ± 0.70 | 11.2 ± 5.60 |
|  | 1Bx7 | 169.8 ± 2.97 | 39.5 ± 8.33 |
|  | 1Dx2.2 | 281.7 ± 4.40 | 25.6 ± 3.28 |
| Anbaek | 1Dy12 | 125.0 ± 0.40 | 13.2 ± 3.25 |
|  | 1By9 | 129.9 ± 2.21 | 19.6 ± 1.26 |
|  | 1Bx7 | 175.9 ± 4.95 | 31.7 ± 2.17 |
|  | 1Dx2 | 229.0 ± 2.15 | 35.5 ± 2.78 |
| Joeun | 1Dy12 | 123.2 ± 1.85 | 13.4 ± 8.36 |
|  | 1By16 | 141.4 ± 1.75 | 21.8 ± 1.38 |
|  | 1Bx13 | 171.7 ± 2.60 | 45.4 ± 7.12 |
|  | 1Dx2.2 | 278.5 ± 4.85 | 19.4 ± 0.13 |
| Joongmo2008 | 10&18 | 137.4 ± 0.12 | 38.1 ± 1.22 |
|  | 1Bx17 | 159.6 ± 0.33 | 29.7 ± 0.86 |
|  | 1Dx5 | 221.5 ± 0.78 | 32.2 ± 0.66 |
| Vesna | 8&10 | 134.3 ± 1.27 | 20.4 ± 1.28 |
|  | 1Bx7^OE^ | 169.4 ± 1.20 | 45.3 ± 2.71 |
|  | 1Ax1 | 202.6 ± 1.93 | 9.5 ± 1.12 |
|  | 1Dx5 | 213.9 ± 2.38 | 24.8 ± 1.06 |

Table S2. Molecular weight of HMW-GS for 8&10 identification by Lab-on-a-chip. Molecular weight was automatically determined by the 2100 Expert program based on molecular size marker. Values are the mean (kDa) ± SD.

| HMW-GS subunit | Saekeumgang | Chapingo | Hanback |
| --- | --- | --- | --- |
| 1Ax2* | 194.9 ± 3.75 | 194.3 ± 2.86 | 191.8 ± 0.64 |
| 1Dx5 | - | 214.8 ± 3.49 | 209.2 ± 0.94 |
| 1Dx2.2 | 278.7 ± 5.80 | - | - |
| 1Bx7 | 169.6 ± 3.30 | 168.3 ± 1.70 | 166.7 ± 0.62 |
| 1By8 | 133.7 ± 1.55 | - | - |
| 1By9 | - | 127.2 ± 0.97 | - |
| 8&10 | - | - | 132.7 ± 0.45 |
| 1Dy10 | - | 134.2 ± 1.25 | - |
| 1Dy12 | 122.7 ± 1.40 | - | - |

Table S3. Molecular weight of HMW-GS for 10&18 identification by Lab-on-a-chip. Molecular weight was automatically determined by the 2100 Expert program based on molecular size marker. Values are the mean (kDa) ± SD.

| HMW-GS subunit | Chapingo | Garnet | Vesna | MK4060 |
| --- | --- | --- | --- | --- |
| 1Ax1 | - | - | 202.6 ± 1.93 | - |
| 1Ax2* | 194.3 ± 2.86 | 191.2 ± 1.73 | - | 196.5 ± 0.90 |
| 1Bx7 | 168.3 ± 1.70 | - | - | - |
| 1Bx17 | - | 154.3 ± 1.73 | - | 158.0 ± 0.97 |
| 1Bx7^OE^ | - | - | 169.4 ± 1.20 | - |
| 1By9 | 127.2 ± 0.97 | - | - | - |
| 8&10 | - | - | 134.3 ± 1.27 | - |
| 10&18 | - | 133.6 ± 0.88 | - | 136.3 ± 0.21 |
| 1Dx5 | 214.8 ± 3.49 | 211.3 ± 2.88 | 213.9 ± 2.38 | 217.0 ± 1.51 |
| 1Dy10 | 134.2 ± 1.25 | - | - | - |

Table S4. Molecular weight (MW) and relative protein quantity (RQ) of HMW-GS for 7^OE^ identification by Lab-on-a-chip. MW and RQ was analyzed by three independent experiments. Values are the mean ± SD.

| Variety Name | HMW-GS subunit | MW(kDa) | RQ(%) |
| --- | --- | --- | --- |
| Jokyung | 8&10 | 133.0 ± 0.54 | 36.6 ± 3.32 |
|  | 1Bx7 | 167.3 ± 0.73 | 23.8 ± 1.07 |
|  | 1Ax1 | 200.6 ± 0.33 | 9.8 ± 2.26 |
|  | 1Dx5 | 210.1 ± 0.29 | 29.8 ± 1.17 |
| MK2686 | 8&10 | 134.6 ± 1.64 | 33.6 ± 1.94 |
|  | 1Bx7 | 170.8 ± 2.50 | 23.6 ± 1.52 |
|  | 1Ax1 | 204.5 ± 3.44 | 10.6 ± 1.31 |
|  | 1Dx5 | 215.7 ± 4.05 | 32.2 ± 0.88 |
| Vesna | 8&10 | 134.3 ± 1.27 | 20.4 ± 1.28 |
|  | 1Bx7^OE^ | 169.4 ± 1.20 | 45.3 ± 2.71 |
|  | 1Ax1 | 202.6 ± 1.93 | 9.5 ± 1.12 |
|  | 1Dx5 | 213.9 ± 2.38 | 24.8 ± 1.06 |
| MK2567 | 8&10 | 134.0 ± 3.59 | 20.3 ± 0.77 |
|  | 1Bx7^OE^ | 169.3 ± 5.65 | 45.4 ± 3.72 |
|  | 1Ax1 | 202.8 ± 5.44 | 9.1 ± 1.57 |
|  | 1Dx5 | 213.9 ± 6.84 | 25.1 ± 2.37 |
| Sammartinara | 8&10 | 134.0 ± 0.75 | 19.3 ± 2.11 |
|  | 1Bx7^OE^ | 169.3 ± 0.92 | 47.2 ± 6.71 |
|  | 1Ax1 | 201.5 ± 1.40 | 6.6 ± 3.31 |
|  | 1Dx5 | 212.4 ± 0.98 | 26.9 ± 1.78 |
| Jasen | 8&10 | 133.9 ± 0.70 | 23.0 ± 2.13 |
|  | 1Bx7^OE^ | 168.4 ± 1.37 | 49.0 ± 7.66 |
|  | 1Ax1 | 201.2 ± 0.84 | 5.2 ± 2.81 |
|  | 1Dx5 | 212.0 ± 0.36 | 22.9 ± 2.82 |
| Keas | 8&10 | 134.8 ± 1.68 | 19.0 ± 5.02 |
|  | 1Bx7^OE^ | 170.3 ± 2.30 | 55.7 ± 9.60 |
|  | 1Ax1 | 203.7 ± 3.50 | 6.2 ± 2.56 |
|  | 1Dx5 | 214.9 ± 4.41 | 19.1 ± 2.18 |
| Safedak | 8&10 | 133.8 ± 1.89 | 16.6 ± 2.11 |
|  | 1Bx7^OE^ | 169.7 ± 2.85 | 52.6 ± 7.73 |
|  | 1Ax1 | 203.0 ± 1.90 | 6.0 ± 3.52 |
|  | 1Dx5 | 213.9 ± 3.52 | 24.9 ± 2.61 |


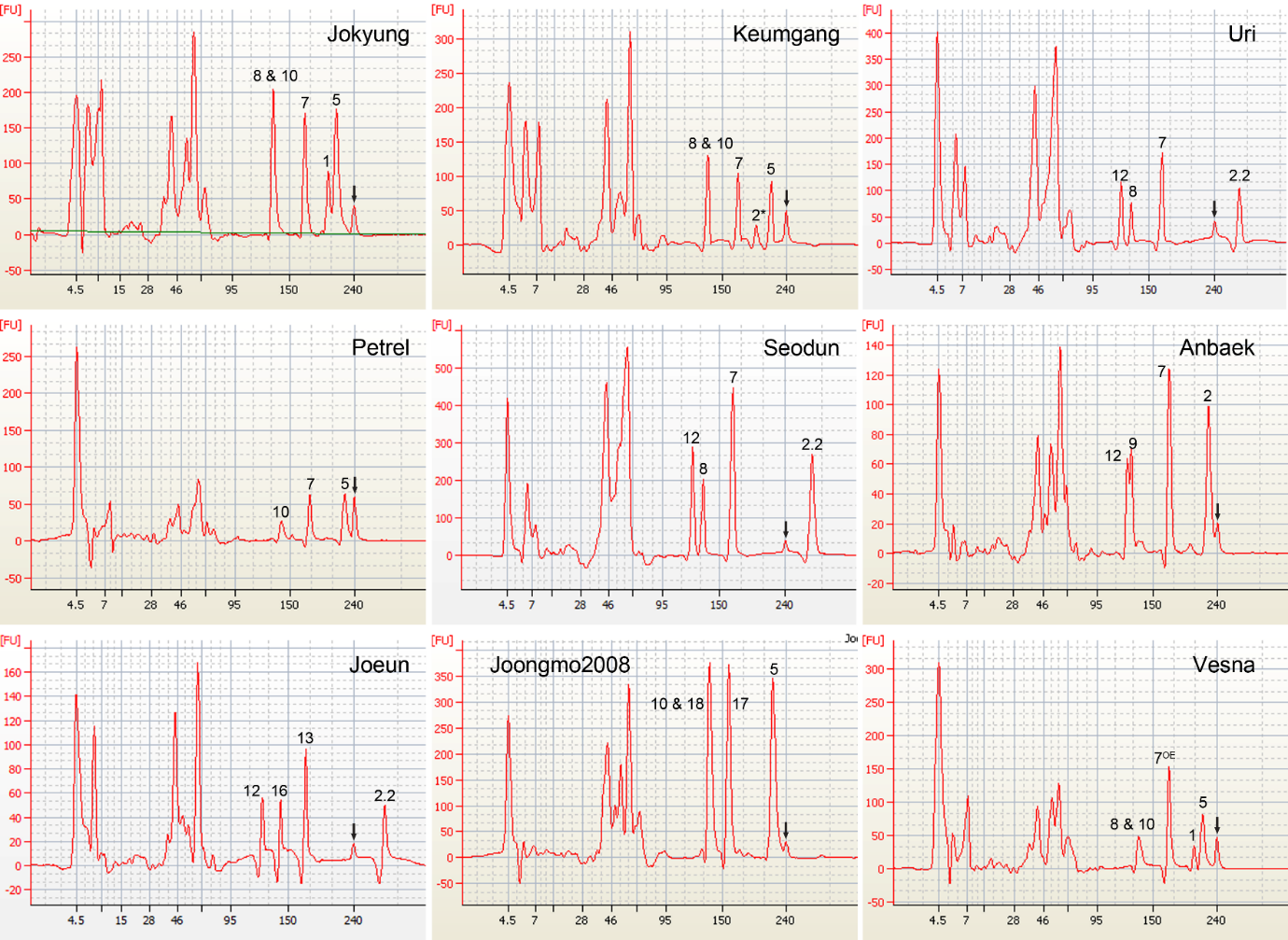


Figure S1. The electropherogram of standard varieties on Lab-on-a-chip. The numbers on the electropherogram mean each HMW-GS subunit such as 5 is 1Dx5 and 1 is 1Ax1.


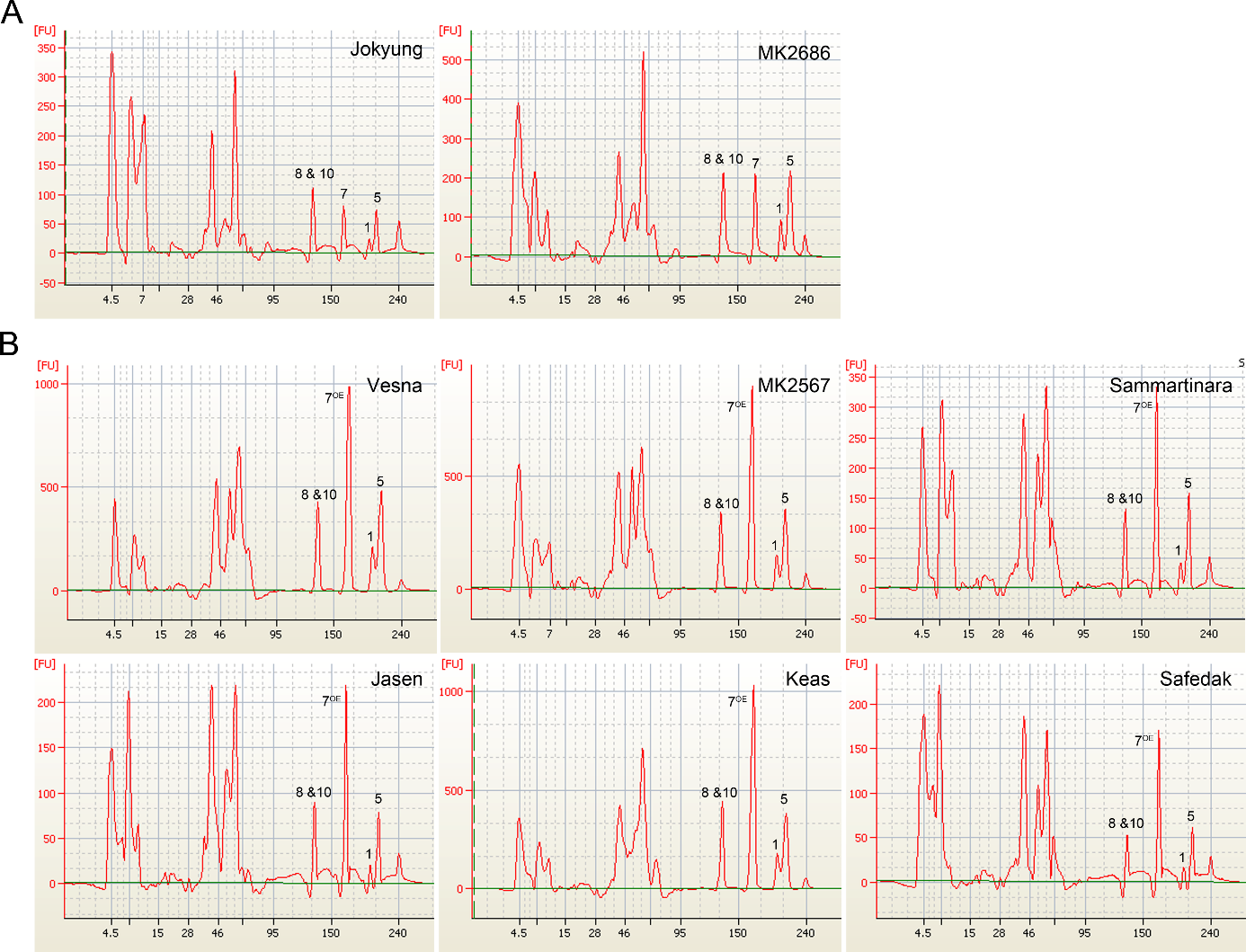


Figure S2. Comparison of electropherogram of 7^OE^ subunit by Lab-on-a-chip. (a) The electropherogram of Jokyung and MK2686 harboring 1Bx7. (b) The electropherogram of varieties harboring 1Bx7^OE^. The numbers on the electropherogram mean each HMW-GS subunit such as 7 is 1Bx7 and 7^OE^ is 1Bx7^OE^.
